# The Modern Diagnosis and Treatment of Diseases of the Stomach

**Published:** 1904-04-30

**Authors:** W. Soltau Fenwick

**Affiliations:** Physician to the London Temperance Hospital, and to the Evelina Hospital for Sick Children.


### The Modern Diagnosis and Treatment of Diseases of the Stomach.

By W. SOLTAU FENWICK, M.D., M.R.C.P., Physician to the London Temperance Hospital, and to the Evelina Hospital for Sick Children.

During the course of the last five-and-twenty years our knowledge of the morbid processes of digestion has undergone a radical change. Up to that time attention had been solely directed to the discovery of microscopical changes in the stomach after death which might account for the symptoms observed during life; and, consequently, most of the gastric complaints which came under the notice of the clinician were attributed to inflammation, ulceration, or to atrophy of the gastric mucous membrane. In 1879, however, Van der Velden drew attention to the fact, previously noted by Prout, that the hydrochloric constituent of the gastric juice disappeared in cases of cancer of the organ; and although the subject attracted little notice at the time, the subsequent introduction of the stomach pump for the treatment of gastrectasis ultimately led to the systematic examination of the contents of the stomach in the various diseases which affect the digestive organs. These investigations in their turn led to remarkable improvements in the technique of gastric analysis and to the introduction of several methods by means of which the size and motorial activity of the stomach could be accurately estimated. Unfortunately, like many other innovations in the science of medicine, the subject of gastric chemistry awakened a degree of enthusiasm which threatened to annihilate the results of experience and common sense, for the disciples of the new school not only claimed that the diagnostic inferences to be drawn from an analysis of the contents of the stomach were practically infallible, but that they rendered all former methods of examination unnecessary. Further experience, however, has shown that the era of infallible diagnosis has not yet dawned, and that the chemistry of digestion, although of undoubted value as confirmatory evidence of disease, must remain subordinate, at any rate for the time, to the methods of physical investigation. In the systematic examination of the stomach, use is made of such methods as inspection, palpation, percussion, and auscultation, as are employed in the investigation of other organs of the body, and of a few which are peculiar to the viscus in question, namely artificial inflation, illumination, and the introduction of a tube.

(1) *Inspection.*—The general aspect of the patient often affords a valuable clue to the nature of his disease, as well as to its extent and rapidity of progress. Loss of energy, physical enfeeblement and mental depression are characteristic features of carcinoma, even from its earliest stage, and are rarely encountered in any other form of gastric disease, however severe the local symptoms may be. The colour of the skin and mucous membranes is another point which always requires attention. Intense anæmia, accompanied by dyspnœa and debility, when it developes rapidly in a dyspeptic subject, usually indicates hæmorrhage, and if there is no history of the vomiting of blood, the stools must be carefully examined for the presence of melæna. On the other hand, the gradual development of an ashen-grey colour after middle life, associated with progressive debility and by the dilatation of venules over the malar bones, is always suggestive of a neoplasm. The condition of the general nutrition is a matter of the greatest moment. Most dyspeptics complain of loss of flesh, but the facts of importance are whether the loss of weight has been gradual or rapid, to what extent it has occurred and whether it is still progressing. In the elucidation of these points the results of systematic weighing can alone be relied upon, and the condition of the tissues must be investigated to confirm the statements of the patient. In children the skin of the abdomen and of the inner sides of the thighs is the first to show signs of a loss of subcutaneous fat, and becomes loose and inelastic; while in the adult the tissues of the breast are the first to undergo atrophy. A tense elastic skin in these regions, therefore, will at once refute any theory of recent emaciation. Rapid and progressive loss of flesh in young individuals who suffer from dyspepsia more often indicates phthisis or diabetes than a primary disease of the stomach, while after middle life the digestive organs are commonly the seat of the mischief.

The special inspection of the digestive system should commence in the mouth, since a defective condition of the teeth, by interfering with mastication, is often responsible for symptoms of indigestion. Inflammation or ulceration of the gums and post nasal growths sometimes give rise to hæmorrhage, and the subsequent vomiting of the swallowed blood may closely simulate true hæmatemesis. The discovery of a blue line upon the gums often throws an unexpected light upon the causation of severe abdominal pain and vomiting which was apparently of gastric origin.

In order to obtain the best results from inspection of the abdomen the patient should lie upon a couch with his head slightly elevated, and his legs bent over a pillow, and be instructed to breathe slowly and easily. A good light should be allowed to fall upon the body from the side opposite to the examiner. In thin persons who are suffering from dilatation of the stomach, the outlines of the organ can often be seen through the attenuated abdominal wall. In such the fulness of the epigastrium which normally exists is found to be replaced by a transverse furrow or depression, while the umbilical region presents an undue prominence. Still more important is the appearance of visible peristalsis. Under normal circumstances the muscular contractions of the stomach are never observed, but if the pylorus or duodenum is contracted the compensatory hypertrophy of the gastric wall at once becomes evident by a succession of peristaltic waves which slowly roll from left to right across the upper abdomen. This peristalsis differs from that of the colon by its strict limitation to the gastric area, by its comparatively slow rhythm, and by the direction of its course towards, instead of from, the right hypochondrium. The contractions can usually be excited by rubbing or kneading the abdomen or by the application of ice to the gastric region, and the rapidity and prominence of the peristaltic waves are most marked when the stomach is moderately distended with food. It may also be observed that stenosis of the pylorus arising from the cicatrisation of a simple ulcer and accompanied by hyperacidity of the gastric contents is usually accompanied by more active peristalsis than the variety which results from carcinoma. As a rule the increased weight of a dilated stomach tends to dislocate the organ downwards, so that the lesser curvature assumes a vertical position, and the pylorus is dragged towards the median line of the abdomen. This fact explains the frequent discovery of a pyloric tumour far below and to the left of its normal situation. When a gastric tumour is of moderate size it may often be observed moving up and down with the respiration, and should it occupy the upper border of the stomach, its connection with that organ becomes established beyond doubt. In like manner it is often possible to determine by inspection the existence of tumours formed by extension of the primary growth to the liver or the great omentum.

(2) *Palpation.*—For this mode of examination the patient should lie upon his back with the shoulders raised and the knees bent over a cushion so as to ensure as much relaxation as possible of the abdominal parietes. Subsequently he may be turned upon his side or be made to assume the knee-elbow position, in order to allow of an examination of the deeper structures. Palpation may be conducted in two ways, according as it is desired to investigate a large area of the gastric wall or only a localised area of the organ. In the former case the right hand of the practitioner should be placed flat upon the abdomen or slightly bent with its ulnar side downwards, while a series of stroking movements are made with the fingers from above downwards. A little practice will enable the shape and outlines of the stomach to be easily recognised, since the walls of the slightly distended stomach impart a uniform elastic sensation to the fingers which is absent in other parts of the abdomen. Palpation with the tips of the fingers is performed with the hand in a vertical position. Each region of the stomach should be investigated in turn, and particular attention paid to the existence of tenderness, undue resistance, and the presence of a circumscribed tumour. It is never wise to rely upon the results of a single examination, especially if they are negative in character, since the palpability of a gastric tumour varies greatly according to the degree of distension of the stomach and intestines. It is therefore important to examine the viscus both before and after a meal, and should the colon be distended, to empty it by an enema or a full dose of castor oil. Tenderness over the stomach usually indicates an inflammatory condition of its tissues: if it be localised to a small area the possibility of ulceration always exists, while a diffuse tenderness is suggestive of gastritis. Hyperæsthesia of the skin over the stomach often accompanies a similar disorder of the gastric nerves. If palpation reveals a tumour special attention must be paid to its position, size, consistency, mobility, degree of sensibility and attachments. There are three tumours which are particularly apt to be mistaken for those due to gastric cancer. First, all gastric diseases being accompanied by constipation, fæcal accumulations are frequently met with in the transverse colon. Careful examination, however, will show that a tumour of this character is more movable and less tender than a cancerous mass, that it is situated below the stomach, that it varies in size from time to time, and that it can be removed by the administration of a large enema or of suitable aperients. Secondly, in some emaciated people who suffer from gastroptosis the pancreas may be felt above the level of the navel. This form of tumour, unlike one attached to the stomach, is immovable and can be shown to lie above the upper margin of the dislocated stomach, Moreover, when the latter organ is artificially inflated, the pancreas ceases to be felt. Thirdly, a lymphatic gland that is situated in the gastrocolic omentum about the centre of the great curvature sometimes becomes sufficiently enlarged in cases of gastritis to be distinctly palpable. The form and attachments of this small tumour, with the knowledge of its occurrence, are usually sufficient to prevent an erroneous diagnosis. Neoplasms of the stomach are most common at the pylorus, and in the vicinity of the lesser curvature, but occasionally the whole of the organ is converted into a cancerous mass. A pyloric growth is usually to be felt above and slightly to the right of the navel, but if there are no adhesions between it and the liver the increased weight of the dilated stomach may drag the pylorus downwards and inwards, so that the tumour is found to be located in the lower part of the abdomen. Cancer of the lesser curvature forms an elongated mass along the upper border of the stomach which is difficult to feel when the organ is empty, while a tumour of the posterior wall can rarely be detected if the stomach is full. Girls who habitually bite or suck their hair sometimes develop a large tumour in the upper part of the abdomen, owing to the accumulation of hair in the stomach. These tumours resemble a kidney in size and shape, but they are very movable, not tender, and exist for many years. In certain cases of gastric cancer, secondary deposits occur in the subcutaneous tissue of the abdominal wall and in the linea alba. The former appear as small nodules at or around the umbilicus, which exhibit a rapid growth, and become adherent to the skin; while the latter occur in the form of an indurated cord which extends from the umbilicus to the pubes in the median line of the abdomen. Occasionally the lymphatic glands above the left clavicle become enlarged from a cancerous infection of the thoracic duct.

(3) *Percussion.*—This method by itself is seldom of any value in the determination of the outlines of the stomach, owing to the difficulty of distinguishing the gastric note from that of the colon and the frequency with which a diseased stomach becomes retracted beneath the ribs and covered by the bowel. The splashing sound which may be elicited by percussion over a stomach containing gas and liquid is sometimes of value in determining the lower border of the viscus. The plan adopted is to tap the abdominal wall with the finger from above downwards and to mark the skin with a pencil at the points where the splash ceases to be heard. The mere existence of a splash, however, has little or no significance, since it can be obtained in any healthy individual who has recently swallowed a moderate quantity of liquid. It is not only when it is elicited in the early morning or several hours after a meal that it indicates insufficiency or atony of the stomach.

(4) *Gastrodiaphany.*—The outlines of the stomach may be rendered visible by an electric illumination of the organ. The apparatus consists of an elastic tube which carries an incandescent light at its lower end. The stomach is filled with water and the tube is inserted in the ordinary way, and the light switched on. When the abdomen is examined in a dark room a luminous area is visible on the anterior wall, which corresponds in outline and position to the upper and lateral boundaries of the stomach. This method has only two points in its favour: it often assists in the location of the upper curvature of the stomach, and may aid the differentiation of gastroptosis from gastric dilatation.

(5) *Gastroscopy* aims at the illumination of the interior of the stomach so that the condition of its mucous surface may be studied by the naked eye. The instrument is similar in mechanism to the cystoscope, and the picture of the inner surface of the stomach is reflected through several prisms. It will be readily understood, however, that the manipulation of this instrument must always be difficult and very disagreeable to the patient, while the results obtained from it are rarely reliable.

*To Determine the Size of the Stomach.*—The results obtained by inspection and palpation may be amplified and corrected by several methods which lay claim to a greater degree of accuracy. *Artificial inflation* with carbonic acid gas is a favourite procedure with many practitioners who aim at rendering the outlines of the organ visible upon inspection. With this object from 40 to 60 grains of tartaric acid dissolved in six ounces of water are administered to the patient and followed immediately by a similar dose of bicarbonate of sodium. A rapid evolution of gas takes place from the interaction of the two substances, and the stomach becomes greatly inflated. If the patient is thin a large swelling becomes visible in the region of the stomach, but if the abdominal wall is thickly covered by fat, palpation is required to determine the position of the distended viscus. Moreover, the existence of incontinency of the pylorus defeats the object of the procedure by allowing the gas to escape immediately into the intestines. The chief value of the method is to determine the existence of an hour-glass stricture and the attachment of tumours connected with the viscus. If the former condition exists the peculiar shape of the stomach can often be observed when it is distended with gas, while the alteration in the position and the palpability of a tumour, which occurs after inflation, is a great aid in distinguishing between growths of the stomach and those of neighbouring organs and in the detection of adhesions. Some authorities prefer to insert a soft tube into the stomach and to pump air into the organ by means of a hand bellows, but this method is as unnecessary as it is disagreeable. Neither method of gastric inflation should be practised if ulcer or cancer of the stomach is suspected, and great care should be exercised whenever there are signs of cardiac disease or emphysema, since the pressure exerted upon the diaphragm by the distended organ is apt in such case to occasion syncope. The most convenient means of determining the size and situation of the stomach is a combination of percussion and auscultation (auscultatory percussion). This is performed in the following manner: Half a pint or more of effervescent soda-water is administered to the patient with the view of procuring moderate distension of the stomach, and he is then directed to lie upon his back with his head and shoulders slightly elevated. The examiner places the end of a stethoscope over the epigastrium and then makes a series of sharp taps with the index finger of the right hand upon the abdominal wall along lines which radiate from the point of auscultation. As long as percussion is made over a spot where the stomach is in contact with the parietes of the abdomen the shock conveyed to the ear continues of the same intensity; but immediately the finger travels off the gastric area the sound becomes faint and toneless. The points at which this change takes place are marked on the skin with a blue pencil, and the investigation is continued until the entire outline of the viscus is mapped out. This method is not only very accurate in its results, but is also easy to perform, and does not entail any discomfort to the patient. The only point which requires special attention is the application of the stethoscope immediately over the stomach.

*Use of the Stomach Tube.*—A soft indiarubber tube is introduced into the stomach for three purposes: (1) To determine whether there exists any mechanical obstruction in the œsophagus or at the cardiac orifice; (2) to extract a portion of the gastric contents for the purposes of chemical examination; (3) to empty or wash out the stomach.

(1) In order to explore the œsophagus the tube should be of moderate size with an opening about one inch from its point, and should be graduated externally in inches or centimetres. The instrument is gently inserted until its progress becomes firmly arrested, when the scale at the level of the incisor teeth is read off and recorded. The patient is then made to cough, so as to drive any material that may exist above the stricture into the interior of the tube, after which the free extremity is firmly closed with the finger, and the instrument quickly withdrawn. Since the average distance in the adult between the incisor teeth and the cardiac orifice is 16 to 18 inches (40-47 cms.), the site of an obstruction is readily determined by reference to the scale of measurement. The material evacuated varies in amount according to the severity of the stenosis, from one drachm to two fluid ounces or more, and usually consists of milk or undigested food mixed with saliva and mucus. In reaction it is neutral or alkaline, and it exhibits no digestive power upon egg-albumin after acidification with hydrochloric acid. If a morbid growth is present in a state of ulceration a small quantity of grumous matter or bright blood may be observed, while occasionally the matter possesses a fetid odour suggestive of sloughing of the neoplasm. On microscopical examination particles of food, epithelial cells, torulæ, cocci, and bacteria can always be detected, and in some instances pus and blood cells or even fragments of the growth may be recognised.

(2) The activity of the gastric secretion is at all times dependent upon the quantity and quality of the food. Thus, in order to obtain results which are capable of comparison with the normal, it is necessary in every case to administer to the empty stomach a meal, the composition of which is constant and accurately determined, and to evacuate the organ after a definite interval of time. The best meal for this purpose is the breakfast devised by Ewald, which consists of a thick slice of bread and butter (70 grammes) and a breakfast cupful of weak tea. The meal is given at eight o'clock in the morning and the stomach is emptied through a tube an hour afterwards. In most cases the evacuation of the semi-digested material is easily accomplished by instructing the patient to cough as soon as the tube has reached the stomach and to withdraw the material which is thus forced into the instrument, after closing the free extremity with the finger; occasionally, however, it may be necessary to exert suction by means of a small syringe. In healthy persons about 30cc. of semi-liquid material can be obtained by complete evacuation of the stomach one hour after the meal. The mixture has a peculiar odour, like that of raw beef, but if fermentation has been active the smell is sour or disagreeable.

*Filtration.*—The first procedure is to filter the mixture through paper. The filtrate should consist of a limpid, yellow, and slightly opalescent fluid, but under pathological conditions the colour may be green from admixture with bile, or red or brown if blood be present; in such cases also the odour may be sour (lactic acid), pungent (excess of hydrochloric acid, acetic acid), rancid (butyric acid), ethereal, saccharine or offensive. The residue upon the paper consists of such materials as have escaped solution. If digestion has been active the amount is small, and the particles of bread appear swollen and gelatinous; but if the gastric secretion has been deficient the residue is considerable in amount and its several constituents are little altered in appearance.

*Tests for the various free acids.*—*Free hydrochloric acid* is most easily recognised by the reaction described by Günsburg. A few drops of the filtrate are mixed in a porcelain dish with an equal quantity of a solution composed of phloroglucin, 2 parts; vanillin, 1 part; absolute alcohol, 30 parts. If free hydrochloric acid is present, on the application of heat a ring of crimson colour develops round the site of evaporation, the intensity of the colour being proportionate to the degree of acidity. The test is an extremely delicate one and is capable of detecting the acid in the proportion of 1 in 10,000.

*Lactic Acid.*—This acid is readily distinguished by means of the test described by Ueffelmann. To 20 cc. of distilled water containing one drop of the tincture of perchloride of iron 10 cc. of a 4-per-cent, solution of carbolic acid are added. The mixture, which is of an amethyst blue colour, immediately changes to canary yellow on the addition of a trace of lactic acid. The reaction is a very delicate one, but is hindered by the presence of phosphates, alcohol, or an excess of hydrochloric acid.

*Butyric acid.*—This acid produces a turbid brown precipitate with Ueffelmann's test, and possesses a characteristic rancid smell. If the filtrate is extracted with ether and the latter separated and evaporated, the residue will contain the greater part of the butyric acid present. If this residue is dissolved in water, and a few pieces of calcium chloride added to it, the acid will separate in the form of oily drops, which are easily recognised.

*Acetic acid.*—To detect the presence of this acid a small portion of the filtrate is extracted with ether, the ether evaporated, and the residue dissolved in water and carefully neutralised with soda. If any acetate of sodium has been formed the addition of a drop of the solution of perchloride of iron will produce a blood-red colour.

*Tests for the Gastric Ferments.*—1. Pepsin. The presence of pepsin in the gastric contents is evidenced by the power of the filtrate to digest albumin. In order to test this 20 cc. of the filtrate are acidified with hydrochloric acid, and the mixture is placed in a flask containing several small cubes of egg-albumin or some shreds of fibrin, and set aside in a warm place. If the ferment is active the albumin will undergo solution, so that at the end of an hour its bulk will have appreciably diminished. 2. Rennet. This ferment is always present in a gastric juice that contains pepsin. To demonstrate its existence 10 cc. of the filtrate are carefully neutralised and mixed with an equal quantity of sterile milk. After exposure to a temperature of 38° c. for half an hour the milk will have undergone coagulation, while the mixture still retains its neutral or alkaline reaction.

*Tests for Blood.*—Blood sometimes exists in such a minute quantity or has undergone so much chemical change that it cannot be identified with certainty by the naked eye. Under these circumstances some special tests for its presence are usually required, of which the following are the most useful: (1) A small quantity of the filtered contents of the stomach are mixed with about one-third of its volume of glacial acetic acid and extracted with ether. A few cubic centimetres of this acid ethereal extract are mixed with 10 drops of tincture of guaiacum and 20 drops of turpentine. If blood is present the mixture turns a bluish-violet colour; if blood is absent it presents a reddish-brown tint. (2) A small quantity of the filtrate is mixed in a porcelain dish with a few grains of chlorate of potassium and a drop of hydrochloric acid, and gently evaporated. If any blood is present the addition of a dilute solution of ferrocyanide of potassium to the residue produces an intense blue colour.

*Test of the Motor Power of the Stomach.*—Several methods have been invented by which the motor power of the stomach can be determined, but the majority of them possess little practical value. The best test is the time required for the complete propulsion of the gastric contents into the duodenum. In healthy persons who do not partake of food during the night the stomach is found to be empty in the early morning, or at most to contain a few drachms of an acid mucoid fluid. If, however, there exists some mechanical obstruction to the exit of food through the pylorus or upper duodenum an appreciable amount of undigested material may be discovered in the organ in the morning. In like manner the normal stomach will dispose of a test breakfast in two hours, but if its motorial functions are deranged a variable quantity can be extracted after the expiration of that time, the amount of which constitutes a rough guide to the degree of gastric insufficiency.

*Microscopical Examination of the Sediment.*—This sometimes affords valuable evidence of disease of the stomach. (*a*) The condition of the undigested particles of food is significant. Coarse masses of meat, the fibres of which preserve their normal striation, or morsels of bread apparently unaltered by their residence in the stomach, indicate a deficiency of the gastric secretion, while a scanty sediment of altered food is a sign that the digestion has been active. (*b*) Sometimes clumps of epithelial cells, or minute pieces of the gastric mucous membrane, may be observed. The former condition is common in inflammatory affections of the stomach and is of no clinical significance, while the latter points to the existence of small erosions of the inner surface of the organ. In cases of carcinoma small masses of growth are occasionally removed from the stomach and greatly aid the diagnosis of the disease, while in other cases groups of cells which exhibit irregular mitoses may be detected. (*c*) Many varieties of micro-organisms are found in the stomach. If the gastric secretion contains much free hydrochloric acid the thread-fungi are most frequently encountered, while a deficiency of the acid is usually accompanied by an excess of the mold organisms. Fermentation in the stomach is associated with yeast, the cells of which are arranged in numerous colonies and are in active process of germination. Sarcinæ, contrary to the usual belief, are comparatively rare in cancer of the stomach, and also in ulcer, nervous diseases, and in dislocation of the viscus. They are very common, on the other hand, in severe and long-standing atony with dilatation. Oppler and Boas have called attention to the presence of a certain bacillus which they believe is chiefly encountered in cases of gastric cancer. This bacillus is easily recognised even in unstained specimens, and is characterised by its great length and motility. It is always associated with an excess of lactic acid in the gastric contents, and its existence is strongly corroborative of the presence of cancer.

*The Value of Exploration of the Stomach in Diagnosis.*—Although it cannot be said that a chemical examination of the gastric contents affords an infallible clue to the nature of the gastric disorder, the systematic employment of the tube often elicits valuable evidence concerning the existence of disease: (1) Resistance to the passage of the tube may arise from (*a*) muscular spasm, (*b*) an organic stricture, or (*c*) pressure upon the œsophagus from without. (*a*) A spasmodic stricture is chiefly met with in young and hysterical women and in drunkards. The tube may be arrested in the pharynx or at any spot in the œsophagus, the site of the obstruction often varying from day to day. Unlike the rigid resistance that is encountered in a case of organic stricture, the tube often appears to become embedded in or grasped by the substance obstructing it, so that an appreciable difficulty is experienced in an attempt to withdraw it, while in many instances it is found that a continued gentle pressure suddenly overcomes the resistance and permits the instrument to be passed into the stomach without further difficulty. These peculiarities indicate that the obstruction is due to a spasmodic contraction of the muscular coat of the œsophagus, similar to that which is met with in the male urethra. (*b*) An organic stricture of the œsophagus is usually situated at a distance of twelve or eighteen inches from the incisor teeth, or, in other words, either opposite the bifurcation of the trachea or at the cardiac orifice. If the action of corrosive fluids can be eliminated it is almost invariably due to carcinoma. The symptoms and signs of the stricture gradually increase in severity, and the food tends to accumulate in the dilated œsophagus immediately above the site of obstruction. The material extracted by the tube is distinguished from the contents of the stomach by its alkalinity, while the presence of blood or pus or the discovery of particles of the neoplasm by the microscope confirms the suspicion of malignant disease. (*c*) Pressure upon the œsophagus is usually caused by an aneurism of the descending thoracic aorta. In such cases, if the tube has been inadvertently employed, the characteristic bruit or shock of the aneurism can be plainly heard by connecting the free end of the tube with a stethoscope. Examination of the fasting stomach throws considerable light upon the state of its motorial and secretory functions. In health the organ is practically empty in the early morning, but in cases of pyloric obstruction or of severe atony with gastrectasis a quantity of undigested food can be evacuated before the first meal of the day. On the other hand, in those diseases of the stomach which chiefly affect the secretory functions the material removed from the fasting organ is found to consist almost entirely of an apolescent or greenish fluid, which possesses active digestive properties, and contains free hydrochloric acid. This demonstration of a continuous secretion of gastric juice usually indicates simple ulceration of a chronic character in the vicinity of the pylorus, and if it is associated with stagnation of the food points to a contraction of the orifice as a result of the disease. Less frequently the hypersecretion arises from some inflammatory or functional disorder of the gastric mucous membrane. The examination of the contents of the stomach after the administration of a test meal is chiefly important in the differential diagnosis of simple ulcer and carcinoma. In the former disease hyperchloracidity is the rule, and free hydrochloric acid can usually be detected in the filtrate and is often present in excess. Lactic acid fermentation, on the other hand, is rarely active, and the organic acid can hardly ever be detected by Ueffelmann's test. In cancer of the stomach the chronic gastritis which invariably accompanies the growth of the neoplasm diminishes and finally inhibits the secretion of the mineral acid, so that the colour tests for free hydrochloric acid usually fail, while lactic acid is found to be present in large amount. When a simple ulcer undergoes a cancerous metamorphosis the free hydrochloric acid gradually diminishes and is eventually replaced by lactic acid.

The existence of altered blood in the stomach is always of serious import, and if it can be repeatedly demonstrated it usually indicates capillary oozing from a soft vascular growth of the gastric wall. Green bile is often associated with hyperchloracidity, but if the bilious fluid is alkaline in reaction and constantly present in the stomach in the early morning there usually exists stenosis of the duodenum below the biliary papilla. The determination of the pepsin and rennet have little practical value, but should the gastric juice when neutralised with carbonate of sodium be found to exert a digestive action upon albumin and starches, its admixture with pancreatic secretion is probable and indicates an obstruction of the third part of the duodenum. Microscopical examination of the sediment seldom throws much light upon the nature of the gastric complaint, since it is difficult to identify isolated cancer cells with any degree of certainty, and the significance of irregular mitosis is still doubtful. The presence, however, of large numbers of the Oppler-Boas bacilli combined with evidence of lactic acid fermentation is of considerable value as evidence of carcinoma.

*Treatment.* (1) Artificial methods of feeding have to be adopted whenever vomiting is so severe as to preclude the administration of food by the mouth, or where the existence of some prominent symptom, such as hæmorrhage or pain, renders it advisable to give the stomach a period of physiological rest. As a rule advantage may be taken of the absorptive powers of the rectum to maintain the nutrition, and if the method of rectal alimentation is conducted efficiently life may be supported for a considerable period. It was formerly believed that the bowel was only capable of retaining and absorbing a small quantity of nourishment at a time, and accordingly about two fluid ounces were directed to be given every two or three hours. But whatever precautions are taken it is invariably found that such injections are not only very disagreeable to the patient, but soon set up irritation of the bowel which interferes with their retention, and accordingly the method cannot be employed for more than a few days without the risk of exhaustion from inanition. It has long been the writer's custom to feed by the bowel upon an entirely different principle, and the results have been so uniformly excellent that a short sketch of the method may possibly be of value. In the first place peptonised milk only is employed, the yolk of eggs being unnecessary and usually being productive of an unpleasant odour in the sick-room. The bowel is washed out with warm normal salt solution each night or morning, and if any irritability exists a few drops of tincture of opium are added to each douche. The apparatus necessary for the administration of the milk consists of a long indiarubber tube about a quarter of an inch in diameter, to one end of which a glass funnel is attached. The tube is warmed and oiled and gently inserted into the bowel as far as it will conveniently go while the patient lies upon his left side with his buttocks slightly raised upon a pillow. The funnel is fixed to the back of a chair two or three feet above the level of the bed and is constantly replenished with warm peptonised milk, as the fluid gradually finds its way into the intestine. About three-quarters of an hour is usually required to administer 16 ounces of milk in this manner, after which the tube is gently withdrawn. The process is repeated about every six hours, by which means from three to four pints are given every 24 hours. The existence of melæna or diarrhœa are the only conditions which prevent the retention of the milk, and under normal conditions not only is the entire quantity absorbed, but the patient is often found at the end of a week or ten days to have increased in weight. Hunger is rarely experienced, and owing to their comparative infrequency the meals seldom produce rectal irritation or prove troublesome to the patient. In every case, however, the mouth must be kept in a moist and aseptic condition, since the diminished secretion of saliva that ensues from the non-mastication of food is apt to lead to inflammation of the parotid gland.

(2) The dangerous fall of the blood-pressure which ensues from excessive hæmorrhage, and also in some cases of peritonitis, may be relieved by saline transfusions. It was formerly the custom to introduce the fluid directly into the circulation through a vein, but the difficulties of obtaining the apparatus and of performing the operation without experienced help has led to the substitution of other methods. Although it may be advisable to increase the amount of fluid in the circulation after a severe loss of blood, it must never be forgotten that the injection of a large quantity of liquid into the vessels is apt to dislodge the blood-clot which had occluded the ruptured artery, and thus bring about another attack of hæmorrhage. It is, therefore, wiser to replenish the fluid in the circulation slowly rather than raise the blood pressure in an abrupt manner. In some cases the introduction of a pint of saline solution into the rectum in the manner already described is sufficient for the purpose, but as a general rule its injection into the subcutaneous tissue is of greater value. With this object a vessel capable of containing about a quart is filled with normal saline solution (a drachm of common salt to a pint of water) at a temperature of 100° F. Siphonage is then established through a length of indiarubber tubing connected with a Southey's tube, and the latter instrument is inserted into the cellular tissue of the axilla, back or inner side of the thigh. If the reservoir of the solution is placed about one foot above the level of the point of insertion of the tube the fluid gradually but steadily finds its way into the tissues of the body, where it is absorbed without giving rise to swelling of the part. Its rate of influx can be increased or diminished by raising or lowering the vessel. In this way a quart of saline solution can be introduced into the circulation within an hour and a half without the danger of exciting a fresh attack of bleeding.

(3) *Hæmorrhage from the stomach* is of common occurrence in ulcerative diseases of the organ and in cases of obstruction of the portal vein. In genuine hæmatemesis the blood which is vomited is considerable, and repeated at rare intervals; while frequent vomiting of small quantities of blood, except in cases of carcinoma, is usually indicative of hæmorrhage from the mouth cr nose which has found its way into the stomach, and subsequently provoked emesis. In all cases of hæmatemesis strict rest in the recumbent position must be enjoined, and in severe cases food by the mouth must be prohibited, and the nutrition maintained by large rectal injections of peptonised milk administered slowly through a long tube. The pulse affords the best indication of the general condition of the patient; a sudden increase of rapidity being suggestive of renewed hæmorrhage, while a gradual augmentation in the number of beats per minute combined with diminished tension being an evidence of increasing debility and the consequent necessity for more nourishment. If the bleeding continues an ice bag may be placed over the stomach in order to control its muscular contractions, while a small dose of morphine is administered subcutaneously to diminish the excitability of the heart. Of the various astringents which are recommended for the treatment of hæmorrhage, gallic acid is the only one which appears to have any beneficial action, and it should be given in doses of four to six grains, combined with a quarter of a grain of extract of opium, in pill form, every three or four hours. In more severe cases better results are obtained from the use of supra-renal extract. If possible, 30 minims of adrenalin chloride, mixed with half an ounce of water, are administered every two hours, but when this preparation cannot be procured, a decoction may be made from the tabloids of the desiccated supra-renal gland, of a strength of five grains to the ounce, of which two ounces or more are given by the mouth every three hours. Should the hæmorrhage continue, it is often useful to apply ligatures round the upper parts of the arms and thighs, sufficiently tight to arrest the venous but not the arterial circulation. By this means a large quantity of blood accumulates in the extremities, and the diminished pressure in the gastric vessels promotes the coagulation of blood at the seat of disease. Should all these measures fail it may be advisable to invoke surgical aid with the view of opening the stomach and seeking the bleeding vessel.

(4) *Dilatation of the stomach*, due to stenosis of the pylorus, usually arises from a simple or cancerous stricture, or from adhesions to the gall-bladder. In the non-malignant cases the secretion of gastric juice is seldom impaired, so that a solid diet may be allowed while all excess of fluids is prohibited. In cancer, on the other hand, it may be necessary to restrict the patient entirely to peptonised milk. Vomiting, which is the principal feature of the complaint, must be combated partly by maintaining the stomach comparatively free from residual food, and partly by the use of such drugs as control the processes of putrefaction. The former indication is attained by the regular use of the stomach tube combined with lavage. The time which should be chosen for washing out the stomach depends upon the character of the symptoms. At an early stage of the complaint the operation may only be necessary two or three times a week, and is most conveniently performed in the early morning; but at a later stage, when the patient finds himself unable to sleep on account of the nocturnal pain or vomiting, the last meal should be taken not later than six o'clock, and the stomach emptied about 10 p.m. Lavage is most conveniently performed by means of a soft tube and a glass funnel, and about three quarts of warm water containing one grain of bicarbonate of sodium to the ounce are usually required. The use of antiseptics is rarely necessary, and their employment is sometimes dangerous, owing to the rapidity with which they are absorbed. The stomach should be evacuated as far as possible before any water has been used, and not more than half a pint of fluid should ever be introduced at a time. At the end of the operation the washings should be measured in order to make sure that all the water employed has been withdrawn. In certain cases washing out of the stomach is contraindicated on account of the dangers that may arise from it. If ulceration is present, the irritation of the tube, or the sudden distension of the organ with water, is apt to provoke bleeding, or to occasion perforation, and many cases have been recorded in which a fatal issue resulted from one or other of these accidents. When fatty degeneration of the heart, or valvular disease is present, great care is requisite, since the pressure of the distended stomach upon the diaphragm is apt to cause syncope. Lavage should not be lightly undertaken when the pyloric mischief is due to corrosive poisoning, for although pain upon swallowing may be absent, the œsophagus is almost invariably ulcerated, and is liable to perforation even by a soft tube. Lastly, a history of cramps of the arms or legs, suggesting as it does the possibility of tetany, should act as a warning against the use of the tube, as in such cases the mere exploration of the stomach has been known to provoke a fatal spasmodic seizure.

Whenever the emesis cannot be allayed by the regular employment of lavage and gastric antiseptics, the question of surgical interference must be considered. In non-malignant cases the performance of gastro-enterostomy usually affords immediate and permanent relief; but if cancer of the pylorus exists, the benefit is only temporary in character, and by the spread of the disease the artificial opening into the bowel often becomes occluded. Excision of the primary growth is attended by a very high mortality, and rarely, if ever, prevents a recurrence of the mischief. In cases of gastrectasis without pyloric obstruction, careful dieting combined with a course of gastric antiseptics will usually suffice without the employment of lavage. Carbolic acid is always of the greatest value, and may be given either in the form of the glycerine preparation (12 mins.) or as a pill. It should always be combined with the carbonates of sodium and bismuth, and administered about two hours after meals. Resorcin, in doses of five to ten grains, is sometimes useful, as it is pleasant to the taste and non-toxic, or, if they be preferred, the sulphocarbolate of sodium (20 grs.), sulphite of sodium (15 grs.), or izal (three mins. in capsules) may be employed. A saline purgative in the early morning is an invaluable adjunct to the medicinal treatment, a full dose of the artificial Carlsbad salts, of phosphate of sodium, or of the compound mixture of magnesia in hot water being usually the most effective.

(5) *Functional Disorders of the Stomach.*—The medicinal treatment of the different forms of dyspepsia varies according to their nature. In *atonic dyspepsia* there is a general enfeeblement of the muscular coat of the stomach and colon accompanied by a deficient secretion of hydrochloric acid and excessive fermentation of the food. All gastric medication must therefore be combined with careful regulation of the bowels. The treatment should be commenced by a course of gastric antiseptics with alkalies, and the administration each night of an aperient pill containing aloes, podophyllin, or colocynth. As soon as the discomfort after food has subsided an effort should be made to treat the primary cause of the disorder. If anæmia is a marked symptom a small dose of the ammonio-citrate of iron or of the tartrate of iron may be administered after meals, or if these preparations cannot be borne the solution of dialysed iron or Flitwick water may be prescribed. No preparation of iron, however, agrees for long, so that every 10 days it should be omitted in favour of a mixture containing bicarbonate of sodium, taraxacum and rhubarb. A mild mercurial pill each night will often aid the digestion of iron. Should atony of the bowel appear to constitute the chief trouble, an effort should be made to cure the constipation by the administration of a pill each night and morning containing a sufficient dose of aloin and a grain of the exsiccated sulphate of iron. Every fortnight the amount of aloin is diminished and that of the iron increased, until eventually the patient finds that the tonic alone acts as an efficient aperient. It must be remembered that recurrent epistaxis, leucorrhœa, bleeding piles, excessive smoking, masturbation, and a sedentary occupation are often responsible for the maintenance of an atonic condition of the digestive organs, and under these conditions it may be necessary to prescribe a dose of pepsin with hydrochloric acid after each meal for a considerable period after the subsidence of the gastric symptoms. Dyspepsia due to an excessive secretion of hydrochloric acid has to be treated in a different manner. In this disorder the symptoms arise from chronic irritation of the stomach by its hyperacid contents, and accordingly the principal indications are to neutralise the acidity and to control the tendency to excessive secretion. A full dose of the artificial Carlsbad salts, or of the phosphate or tartrate of sodium, given in hot water each morning before breakfast, is the best remedy for the constipation which invariably accompanies this gastric complaint, while a mixture of bicarbonate of sodium, carbonate of bismuth and carbolic acid two hours after the principal meals usually arrests the pain and acidity. In severe cases it may be necessary to add ten minims of the solution of hydrochlorate of morphine to the medicine, or use may be made of the tincture of belladonna or cannabis indica. When the discomfort is chiefly experienced at night and interferes with the sleep, the patient should be advised to drink a tumblerful of milk in which 20 grains of bicarbonate of sodium have been dissolved. If the stomach is dilated owing to the contraction of a chronic ulcer of the pylorus, lavage or gastro-enterostomy may be requisite. Nervous dyspepsia is usually associated with the symptoms of neurasthenia and with the signs of gastrectasis and gastroptosis. A firm belt worn tightly round the waist will at once relieve the sense of dragging and emptiness which is experienced at the epigastrium, and if exhaustion is complained of after an action of the bowels a daily enema of soap and water should be ordered instead of aperient medicines. It is in cases of this description that dilute hydrochloric acid, combined with pepsin, after meals is of such value, and if flatulence is a troublesome symptom a small dose of carbolic acid may be added to the mixture with great benefit. At a later stage three grains of valerianate of zinc administered night and morning after food proves a valuable tonic.

